# Huge intrameniscal cyst successfully treated by open debridement and combined arthroscopic and open repair: a case report

**DOI:** 10.1186/s12891-020-03218-0

**Published:** 2020-03-27

**Authors:** Young-mo Kim, Darryl D. D’Lima, Yong-bum Joo, Il-young Park

**Affiliations:** 1 Department of Orthopedic Surgery, Chungnam National University Hospital, Chungnam National University School of Medicine, Munhwa-dong, Jung-gu, Daejeon, 301-721 South Korea; 2 Shiley Center for Orthopaedic Research and Education at Scripps Clinic, La Jolla, CA 92037 USA

**Keywords:** Intrameniscal cyst, Meniscal tear, Meniscal repair, Arthroscopy, Knee

## Abstract

**Background:**

Meniscal cysts are not uncommon in clinical practice, with reported incidence rates varying from 1 to 22%. Most meniscal cysts are parameniscal cysts, which are created by extravasation of synovial fluid through the meniscal tear into the adjacent soft tissue. In contrast, intrameniscal cysts in which the fluid collects in the meniscus are very rare. We encountered a teenager with a huge intrameniscal cyst accompanied by a small vertical meniscal tear in the red-white zone of the upper surface of the medial meniscus. A literature search revealed no information regarding the appropriate treatment methods and results for this type of lesion.

**Case presentation:**

A 14-year-old boy presented to our outpatient clinic because of right knee pain that had been present for the previous 2 months. The patient participated in Hapkido, but had no specific trauma history. Magnetic resonance imaging revealed a huge intrameniscal cyst located in the central parenchyma of the posteromedial corner of the medial meniscus. In addition, one sagittal slice on MRI revealed a vertical tear in the red-white zone of the upper surface of the medial meniscus. The presence of such a tear accompanied by a huge intrameniscal cyst is very unusual. The patient was treated via arthroscopic inside-out meniscal suture repair and open cystic debridement with additional meniscocapsular suturing. During 4 years of magnetic resonance imaging follow-up, the lesion has completely disappeared and the meniscus has successfully recovered its normal form.

**Conclusions:**

Our treatment method may be considered as the first choice for young patients who require surgical treatment for large intrameniscal cysts with accompanying small vertical meniscal tears.

## Background

Meniscal cysts are not uncommon in clinical practice, with reported incidence rates varying from 1 to 22% [1]. Most meniscal cysts are accompanied by meniscal tears [2, 3], and are classified as intrameniscal or parameniscal cysts in accordance with the position at which the cystic fluid collects [2].

Most meniscal cysts are parameniscal cysts, which are created by extravasation of synovial fluid through the meniscal tear into the adjacent soft tissue. The treatment principles for parameniscal cysts are well established, comprising treatment of the meniscal lesion and decompression of the cystic lesion [2].

In contrast, intrameniscal cysts in which the fluid collects in the meniscus are very rare [2]. Intrameniscal cysts form via the check valve mechanism [4]. Local enlargement of the meniscus occurs based on the amount of accumulated fluid, and intrameniscal cysts are generally treated like a meniscal tear [2]. Few studies have evaluated intrameniscal cysts, and so there is insufficient information available regarding the mechanism of development, diagnostic criteria, and treatment methods.

We encountered a teenager with a huge intrameniscal cyst accompanied by a small vertical meniscal tear in the red-white zone of the upper surface of the medial meniscus (MM). However, a literature search revealed no information regarding the appropriate treatment methods and results for this type of lesion. We performed arthroscopic inside-out meniscal suture repair and open cystic debridement with additional meniscocapsular suture. Four years of continuous magnetic resonance imaging (MRI) follow-up confirmed that the lesion was completely resolved, and the meniscus was restored to normal.

Herein, we report the details of this rare case and review the relevant literature.

## Case presentation

A 14-year-old boy presented to our outpatient clinic because of right knee pain that had been present for the previous 2 months. There was persistent pain in the medial side of the right knee, and the pain was worse on knee flexion while weight-bearing. Conservative treatment performed in a local clinic did not improve the symptoms. The patient participated in Hapkido, but had no specific trauma history.

On physical examination, there was medial joint line tenderness. There was no joint effusion or limitation of the range of motion. There were no palpable soft tissue mass lesions on the medial side of the knee.

**Fig. 1:**
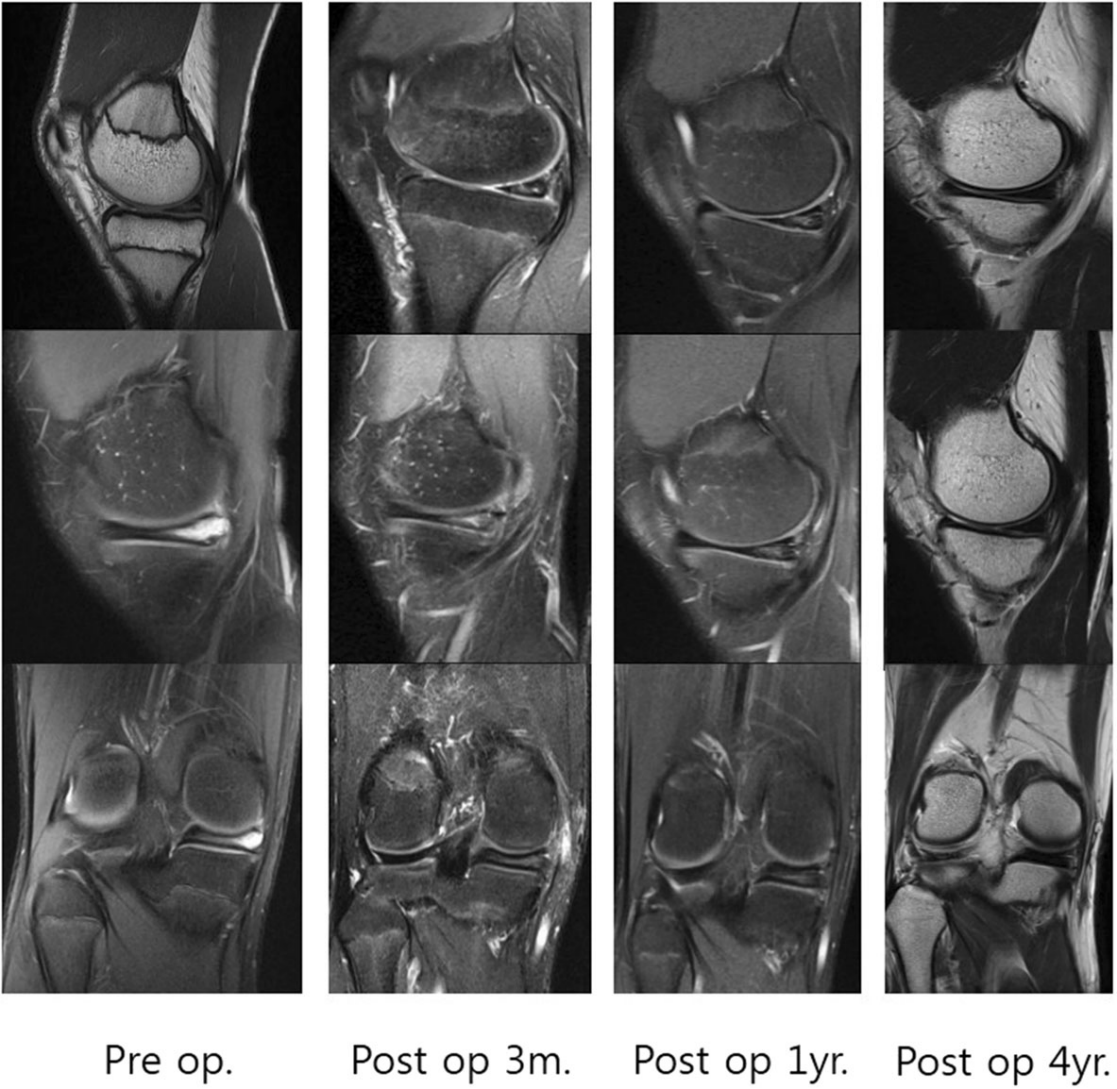
Pre op. Magnetic resonance images showing a huge intrameniscal cyst located in the posteromedial corner of the medial meniscus accompanied by a very thin meniscal parenchymal layer between the cyst and the upper and lower surfaces of the meniscus. Vertical tear of the upper meniscal surface in the red-white zone of the posteromedial corner of the medial meniscus. Post op. Follow-up magnetic resonance images taken about 3 months, 1 year, 4 years postoperatively showing that the high signal intensity where the meniscal cyst was slowly disappeared over time. Four year postoperatively showing a normalized meniscus

**Fig. 2:**
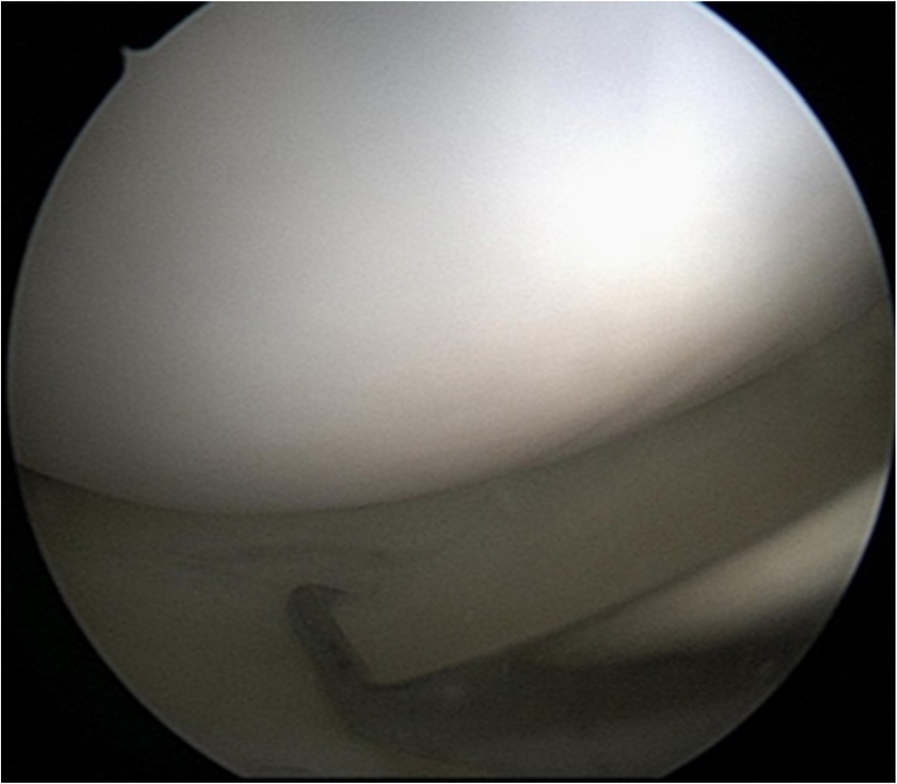
Arthroscopic examination showing a small vertical longitudinal tear in the red-white zone of the upper surface of the posteromedial portion of the medial meniscus

There were no abnormal findings on plain radiography. MRI revealed a huge intrameniscal cyst located in the central parenchyma of the posteromedial corner of the MM, and a very thin meniscal parenchymal layer between the cyst and the upper and lower surfaces of the meniscus (Fig. 1, Pre op.). However, it could not be clearly identified whether the thinness of the parenchyma was caused by a pressure effect or a loss of degenerated meniscal substance due to cystic fluid. In addition, one sagittal slice on MRI revealed a vertical tear in the red-white zone of the upper surface of the MM (Fig. 1, Pre op.). Therefore, the tear was classified as a stage 2B meniscal tear using the Stoller and Crues 3-stage MRI classification of meniscal degeneration. The presence of such a tear accompanied by a huge intrameniscal cyst is very unusual [5–7]. Arthroscopy was performed to identify and treat the lesions identified on MRI as an intrameniscal cystic lesion and suspected meniscal tear. On the posteromedial portion of the MM, arthroscopy revealed a small vertical longitudinal meniscal tear in the red-white zone of the upper surface of the MM (Fig. 2). Arthroscopic probing also confirmed the presence of a cystic lesion inside the meniscus.

**Fig. 3:**
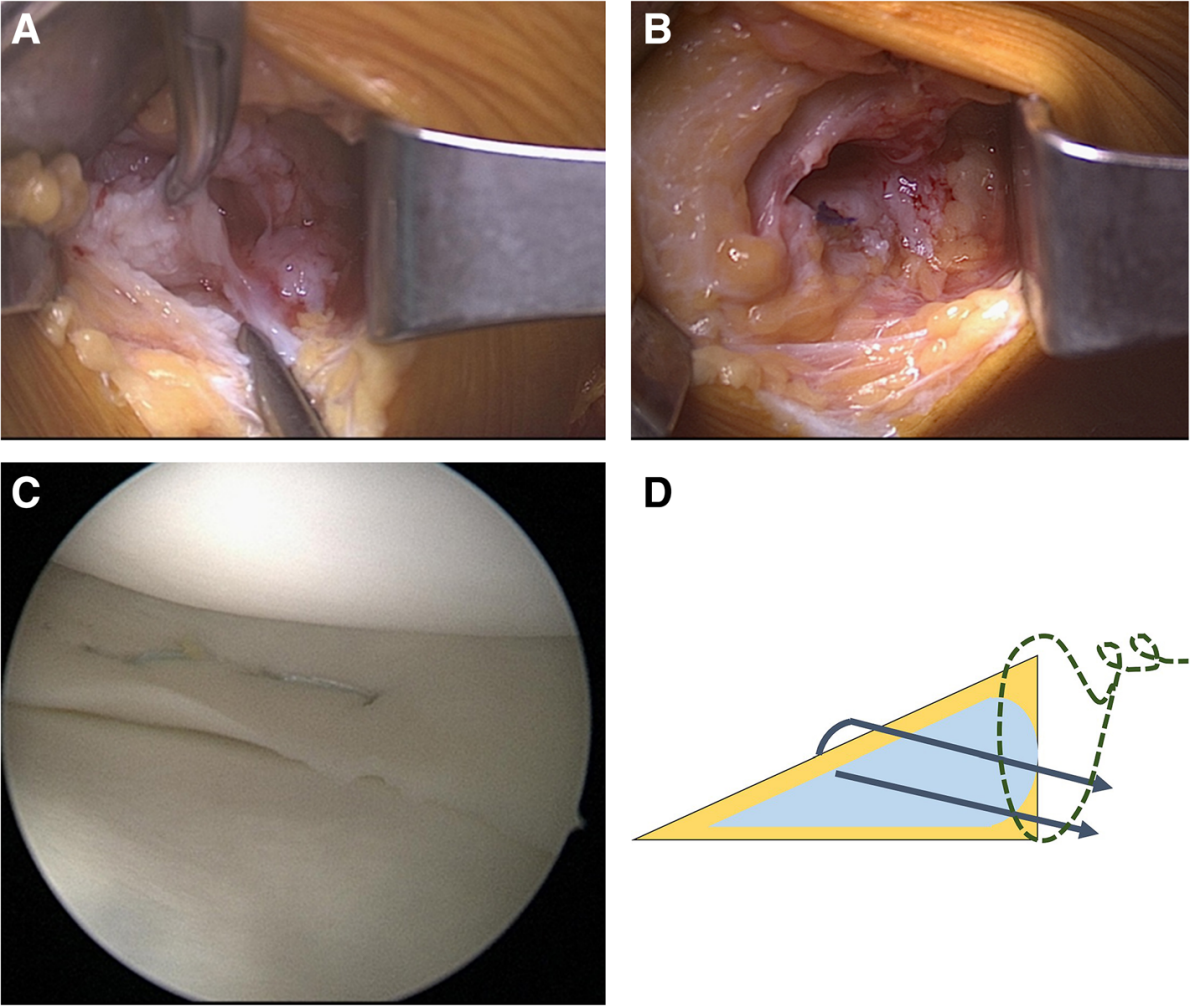
**a** The meniscal cyst is exposed through the skin incision and soft tissue dissection. **b** Open meniscal repair with vertical sutures using 0 vicryl after cyst wall debridement using a meniscal rasp and a curette through an open incision. **c** The placement of two inside-out horizontal meniscal sutures. **d** Simplified illustration showing the suture repair performed for the intrameniscal cyst and associated meniscal tear of the upper surface of the medial meniscus

As the patient was only 14 years old, we decided to preserve the meniscus through suturing as the primary operation. However, because the lesions were chronic, it was expected that simple arthroscopic repair alone would not achieve a successful outcome. Thus, we decided to also perform open meniscal repair with cystic debridement by directly accessing the intrameniscal cyst through a skin incision. A small vertical skin incision was made over the medial joint line, and the subcutaneous tissue of the posteromedial corner of the knee was dissected to expose the posteromedial capsule behind the medial collateral ligament. Under arthroscopic translumination assistance, the joint capsule was transversely incised just proximal to the upper surface of the MM to prevent iatrogenic meniscal injury. The upper surface of the MM was identified, and the meniscal cyst was exposed through an additional small transverse incision of the meniscocapsular junction (Fig. 3a). A meniscal rasp and a curette were used to debride the cyst wall through the open incision. Open meniscal repair was performed with vertical sutures using 0 vicryl (Polyglactin 910; Ethicon, Somerville, NJ, USA) to close the surgically opened intrameniscal cyst (Fig. 3b). Two inside-out horizontal meniscal sutures were placed using no. 2–0 FiberWire meniscal repair needles (Arthrex, Naples, FL, USA) and zone-specific cannulas (Linvatec, ConMed, Largo, FL) (Fig. 3c). Figure 3d shows a simplified illustration of the suture repairs performed for the intrameniscal cyst and the tear of the upper surface of the MM. Biopsy was not performed.

Range of motion exercise was started immediately after the surgery. The patient was only permitted to walk with crutches to restrict weight-bearing until 6 weeks postoperatively. Weight-bearing was then gradually increased. Full weight-bearing was permitted from 3 months postoperatively. Return to daily sports activity was allowed at 6 months postoperatively. The patient was regularly followed up, and consented to undergo MRI at 3 months, 6 months, 1 year, 2 years, 3 years, and 4 years postoperatively. Over time, the high signal intensity where the meniscal cyst was located slowly disappeared (Fig. 1, Post op.)

At 4 years postoperatively, MRI showed a normal meniscus (Fig. 1, Post op.) There was no recurrence of symptoms, and the International Knee Documentation Committee 2000 subjective knee score was significantly improved from 42 points preoperatively to 93 points at 4 years postoperatively. The patient reported that he did not feel any discomfort during daily life and exercise.

## Discussion and conclusions

Meniscal cysts may present clinically as a painful mass [2]. The reported incidence of meniscal cysts varies from 1 to 22% [1], but they are not uncommon in clinical practice. There are some literatures reporting meniscal cyst in children and adolescents (Table 1) [4, 8–14].

**Table 1:** literatures reporting meniscal cyst in children and adolescents

Year	Author	No. of patients aged< 20 yr
1929	Cambell and Mitchell [8]	1
1965	Becton and Young [9]	9
1975	Jaffres [10]	1
1980	Schuldt and Wolfe [11]	5
1988	Stern and Hallel [12]	1
1992	VanderWilde and Peterson [13]	11
2005	Lippe [14]	1
2014	Imamura [4]	1

Meniscal cysts are classified as intrameniscal or parameniscal in accordance with the location of cystic fluid accumulation [2]. In intrameniscal cysts, the fluid accumulates in the meniscal parenchyma, with or without focal enlargement of the meniscus. Parameniscal cysts comprise fluid that has extravasated through a meniscal tear into the extraarticular surrounding soft tissue [2]. Therefore, parameniscal cysts usually present peripherally as a subcutaneous lump with a positive Pisani’s sign [1].

Several recent studies have reported an 100% association between meniscal cysts and existing meniscal tears [3]. Meniscal cysts are also reportedly closely related to meniscal surgeries, and meniscal cysts are observed in 1 to 8% of MRI examinations performed after meniscectomy or meniscal repair [4]. A study on the MRI appearance of meniscal cysts reported that only 5% of meniscal cysts are totally intrameniscal, whereas the remaining 95% are parameniscal [2]. As parameniscal cysts are more common than intrameniscal cysts, the etiology and treatment principles for parameniscal cysts are well established. Generally, successful treatment of parameniscal cysts requires intra-articular surgery to decompress the cyst and address the meniscal pathology [2].

In contrast, intrameniscal cysts are relatively rare [1]. Thus, the etiology, diagnostic criteria, and treatment methods of intrameniscal cyst are unclarified [4, 15–17]. One previous study reported that intrameniscal cysts are treated like meniscus tears [2]. However, our literature search did not retrieve any reports detailing the successful treatment process and results for intrameniscal cysts.

Peetrons et al. [15] suggested that intrameniscal cysts are caused by the influx of synovial fluid through a check valve made by the meniscal tear. Based on this mechanism, Imamura et al. [4] used arthroscopic check valve release to treat the symptoms of an intrameniscal cyst.

In our case, preoperative MRI revealed that the meniscal parenchyma between the intrameniscal cyst and the upper and lower meniscal surfaces was very thin. Preoperatively, it was unclear whether this thin meniscal parenchyma was due to the pressure effect or the loss of degenerated meniscal substance. Therefore, it was unclear whether meniscal repair was the best treatment until postoperative follow-up MRI was performed. Follow-up MRI confirmed that the meniscus had recovered its normal shape, suggesting that the very thin meniscal parenchyma between the intrameniscal cyst and the upper and lower meniscal surfaces was caused by the increased intrameniscal fluid pressure due to the check valve mechanism. In children, the meniscus has growth potential; it is more cellular and elastic. Hydrostatic pressure is more likely to stretch this kind of tissue, and hence it is not a surprise that the balloon -like growth is sealed inside the meniscus whereas in adults, the meniscus is less elastic and intra-meniscal fluid bursts more easily to surrounding tissue. The small vertical tear in the upper meniscal surface adjacent to the intrameniscal cyst acts as a one-way check valve that causes synovial fluid to flow into the intrameniscal cyst. So these synovial fluids serve as the developmental origin of this cyst. As a result, the pressure increases in the intrameniscal cyst and compresses the surrounding meniscal parenchyma.

The intrameniscal cyst subsequently increases in size and generates an inflow of fluid through the check valve. A continuous cycle of this sequence of processes resulted in the creation of a huge intrameniscal cyst and a very thin meniscal parenchymal layer between the cyst and the upper and lower meniscal surfaces. This cyclic process led to the clinical symptoms in the present case. If MRI is performed while the lesion is advanced, as in the present case, the appearance of the lesion is highly likely to cause confusion regarding the appropriate choice of operative procedure among meniscal repair, meniscectomy, and other methods (including check valve release). Therefore, the present case has great implications and has shown that arthroscopic and open meniscal repair with debridement is a successful treatment method for a huge intrameniscal cyst with an associated small tear of the red-white zone of the upper meniscal surface in a young patient. Although Imamura et al. [4] successfully treated the subjective symptoms of their patient by creating a cystic opening into the joint cavity via check valve release, the disadvantage of this method is that the meniscus was not anatomically restored through repair. In our case, the meniscal tear that acted as a check valve was located in the red-white zone and the patient was a teenager; thus, it was decided that it was inappropriate to perform arthroscopic check valve release alone.

Biedert [16] compared four treatment options for isolated, symptomatic, painful, horizontal, grade 2, medial intrasubstance meniscal tears in 40 patients. The four treatment options were conservative therapy, arthroscopic suture repair with trephination (to create access channels), arthroscopic minimal central resection, and suture repair with an intrameniscal fibrin clot and arthroscopic partial meniscectomy [16]. They concluded that intrasubstance meniscal lesions are best treated by partial meniscectomy, but that arthroscopic suture repair with trephination might give better medium- to long-term results by preserving the meniscal function [16]. Although meniscal repairs have a higher reoperation rate than partial meniscectomies, they are associated with better long-term outcomes, higher clinical scores, and less radiographically detected degeneration [18]. Meniscal repair may avoid the long-term articular cartilage degeneration that results from meniscectomy.

Furthermore, Pujol et al. reported good subjective clinical results after open meniscal repair with torn meniscal tissue debridement for horizontally torn menisci in young patients. They performed open meniscal repair in 21 young patients with horizontal meniscal tears (10 grade 3 tears and 11 grade 2 tears), and reported that 16 patients (84%) were satisfied or very satisfied with the operation and would undergo surgery again if necessary. In addition, meniscal cysts were present in 15 of 21 cases, although they were not subdivided into parameniscal and intrameniscal cysts. Identified meniscal cysts were excised and no recurrence was reported [17].

We considered the present patient’s young age to be more important than any other factor.

In elderly patients with degenerative meniscus, partial meniscectomy by arthroscopic tunneling of the cyst is the gold standard. In our young patient, primary treatment goal was to normalize the meniscal tear and cyst as much as possible through suturing, although there was a substantial possibility that the patient would need further surgery.

The vertical tear of the upper meniscal surface and the intrameniscal cyst were repaired with two arthroscopic inside-out horizontal sutures. To increase the likelihood that the meniscal lesion would heal, the open approach was used to incise the cyst, debride the degenerative tissue of the cystic wall, and perform additional meniscocapsular suturing. In 4 years of continuous MRI follow-up, the intrameniscal cystic lesion and the red-white zone tear of the upper meniscal surface have slowly healed and completely disappeared, and the meniscus has successfully recovered its normal form.

Therefore, we recommend combined arthroscopic and open suture repair with cystic debridement for intrameniscal cystic lesions in young patients.

## Data Availability

This is a case report of a single patient, to protect privacy and respect confidentiality; none of the raw data has been made available in any public repository. The original reports, laboratory studies, imaging studies and outpatient clinic records are retained as per normal procedure within the medical records of our institution.

## Abbreviations

MM: Medial meniscus
MRI: Magnetic resonance imaging
